# A Clinical Encounter With Intrathoracic Extrapulmonary Hydatid Disease

**DOI:** 10.7759/cureus.48496

**Published:** 2023-11-08

**Authors:** Souvik Sarkar, Pankaj Wagh, Ulhas Jadhav, Babaji Ghewade

**Affiliations:** 1 Respiratory Medicine, Jawaharlal Nehru Medical College, Datta Meghe Institute of Higher Education and Research (Deemed to be University), Wardha, IND

**Keywords:** intrathoracic extrapulmonary hydatid cyst, albendazole antihelminthic, echinococcus spp, massive pleural effusion, pulmonary hydatid disease

## Abstract

Intrathoracic extrapulmonary hydatid disease is an uncommon variant of hydatidosis. In this report, we describe a rare case of a 53-year-old female who presented with a left-side massive hydropneumothorax, initially treated as tubercular empyema and later came out to be intrathoracic extrapulmonary hydatid disease, with no signs of primary lesion in the lung. This case was managed with an intercostal drain insertion followed by a thoracoscopic-guided excision of the cyst, which on histopathological examination confirmed the diagnosis. Also, the *Echinococcus* antibody IgG test confirmed the same. The patient was then initiated on oral albendazole which showed a drastic reduction in the intrapleural cysts, but the patient later developed non-resolving pyopneumothorax with a bronchopleural fistula. The patient is being managed conservatively at present with oral albendazole and chest drain and is later advised to undergo decortication surgery of the lung.

## Introduction

Hydatidosis is caused by accidentally ingesting eggs containing the larval stage of dog tapeworm or *Echinococcus granulosus* from infected canines. The lung is the second most common site involved after the liver. Intrathoracic extrapulmonary hydatid disease is an even rare variant of hydatidosis [1]. It has been reported that between 7.4% and 10.5% of intrathoracic hydatid cysts are extrapulmonary [2]. Pleural hydatidosis cases are extremely uncommon but should be taken into account in individuals from endemic areas who may have been exposed to *Echinococcus*. A great recovery can be achieved with the right surgical and antiparasitic procedures. Any kind of hydatidosis can be well managed with antiparasitic therapy, although surgery remains the mainstay of care.

## Case presentation

**Figure 1:**
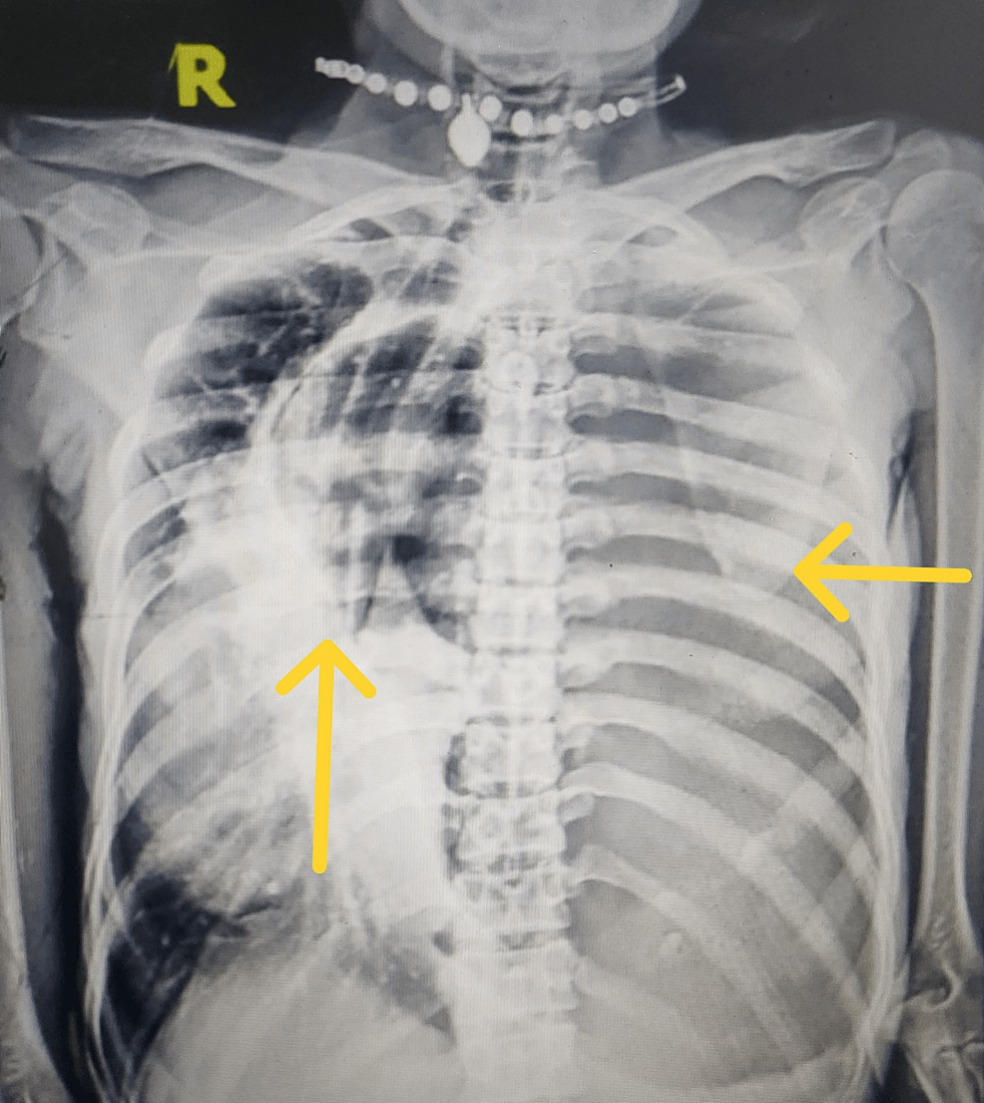
A supine anteroposterior chest X-ray on admission was suggestive of left-side massive hydropneumothorax with a shift of the mediastinum to the right.

A 53-year-old thin-built, female patient farmer by occupation presented with complaints of acute worsening of breathlessness, which she had for two months, along with on-and-off fever associated with chills and rigors. On examination, the patient had a pulse rate of 132 beats per minute and blood pressure of 110 by 60 mm Hg. She was tachypneic with a respiratory rate of 38/min and was maintaining an oxygen saturation of 90% without oxygen support. On auscultation of the chest, her breath sounds were reduced on the left side. A supine, anteroposterior chest X-ray was suggestive of left-side massive hydropneumothorax with a shift of the mediastinum to the right (Figure 1).

**Figure 2:**
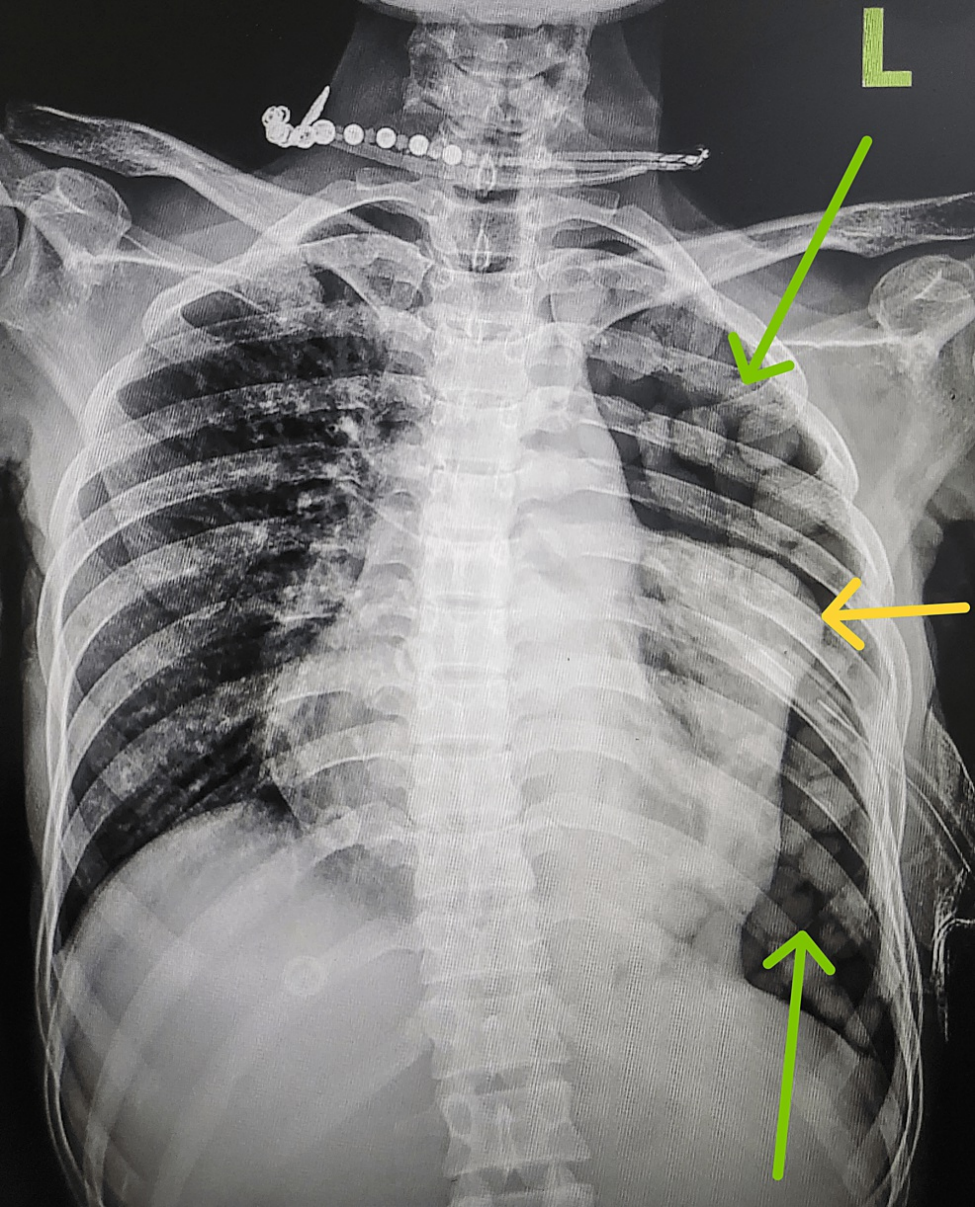
Chest X-ray suggestive of pneumothorax with multiple homogeneous rounded opacities (green arrows) and chest drain in situ in the left hemithorax. The left lung was collapsed (yellow arrow).

Her lab blood workup was unremarkable with a normal eosinophil count. An intercostal drainage (ICD) tube was inserted immediately, and a turbid pleural fluid was drained and sent for investigation. The pleural fluid was neutrophilic exudative with 60% polymorphs, and the adenosine deaminase (ADA) level was 43 IU/L. Acid-fast bacilli smear and cartridge-based nucleic acid amplification test showed absence of *Mycobacterium tuberculosis*. Bacterial and fungal culture showed no growth. Thus, the patient was then initiated on anti-tubercular treatment on the basis of raised ADA. But a follow-up chest X-ray after four days showed left-sided pneumothorax with an atelectatic lung and multiple homogeneous rounded opacities in the left hemithorax (Figure 2).

**Figure 3:**
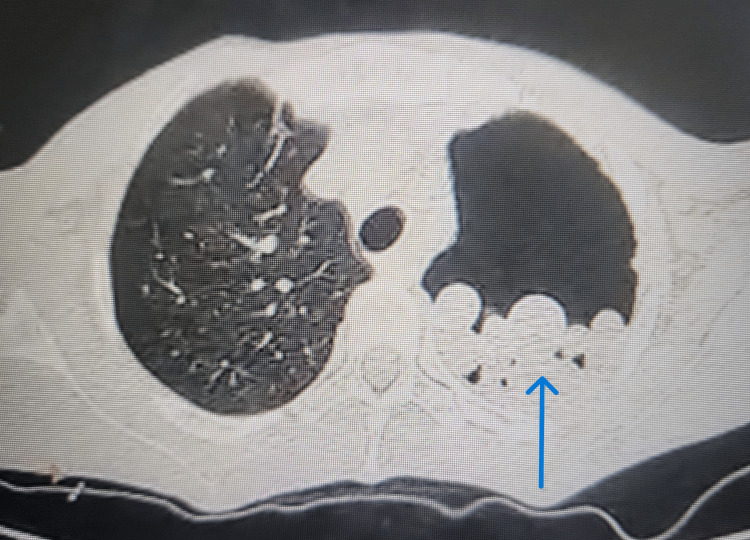
Multiple well-defined homogeneous rounded opacities (blue arrow) seen in the dependent portion of the left pleural cavity with collapse of the lung on the same side.

A contrast CT was also done which revealed multiple well-defined homogeneous rounded opacities seen in the dependent portion of the left pleural cavity with no post-contrast enhancement. The left lung was completely collapsed and the right lung was normal. Few mediastinal lymphadenopathies were noted (Figure 3).

**Figure 4:**
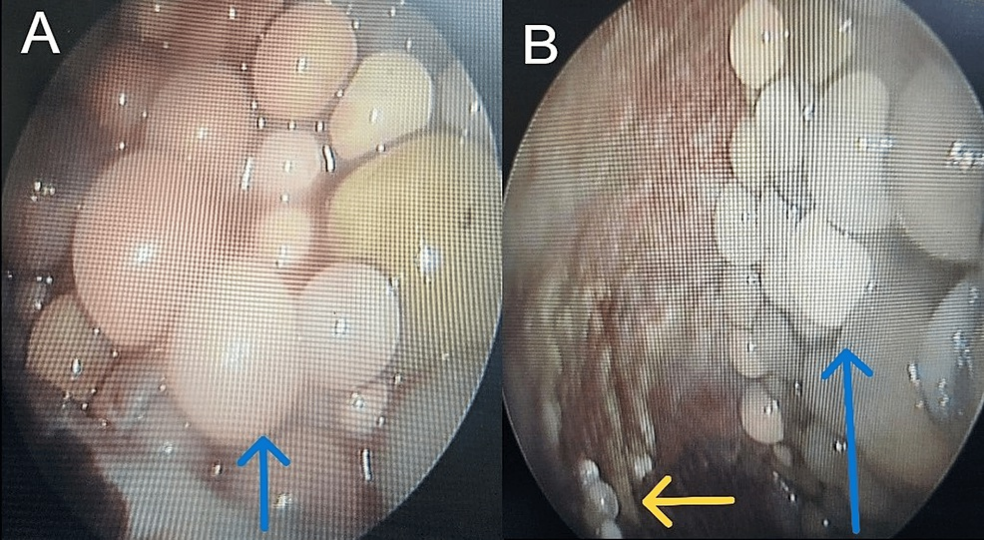
Thoracoscopic images showing multiple cystic nodules of size 1-4 cm diameter in the pleural cavity adhering to the visceral pleura (A) and the parietal pleura (B).

All the radiological findings were suggestive of intrapleural extrapulmonary hydatidosis. A medical thoracoscopy was then performed which showed cystic nodules of varying sizes of 1-4 cm diameter in the pleural cavity adhering to the parietal and visceral pleura (Figure 4).

A cyst was taken from the same and sent for histopathological examination. The histopathology report stated that the section studied from the cyst wall showed histopathological features of the hydatid cyst. However, a serum absolute eosinophil count of the patient was normal (120 cells/cc). The patient was then subjected to serum *Echinococcus granulosus* IgG antibody test which was positive and confirmed our diagnosis of intrapleural extrapulmonary hydatid disease.

**Figure 5:**
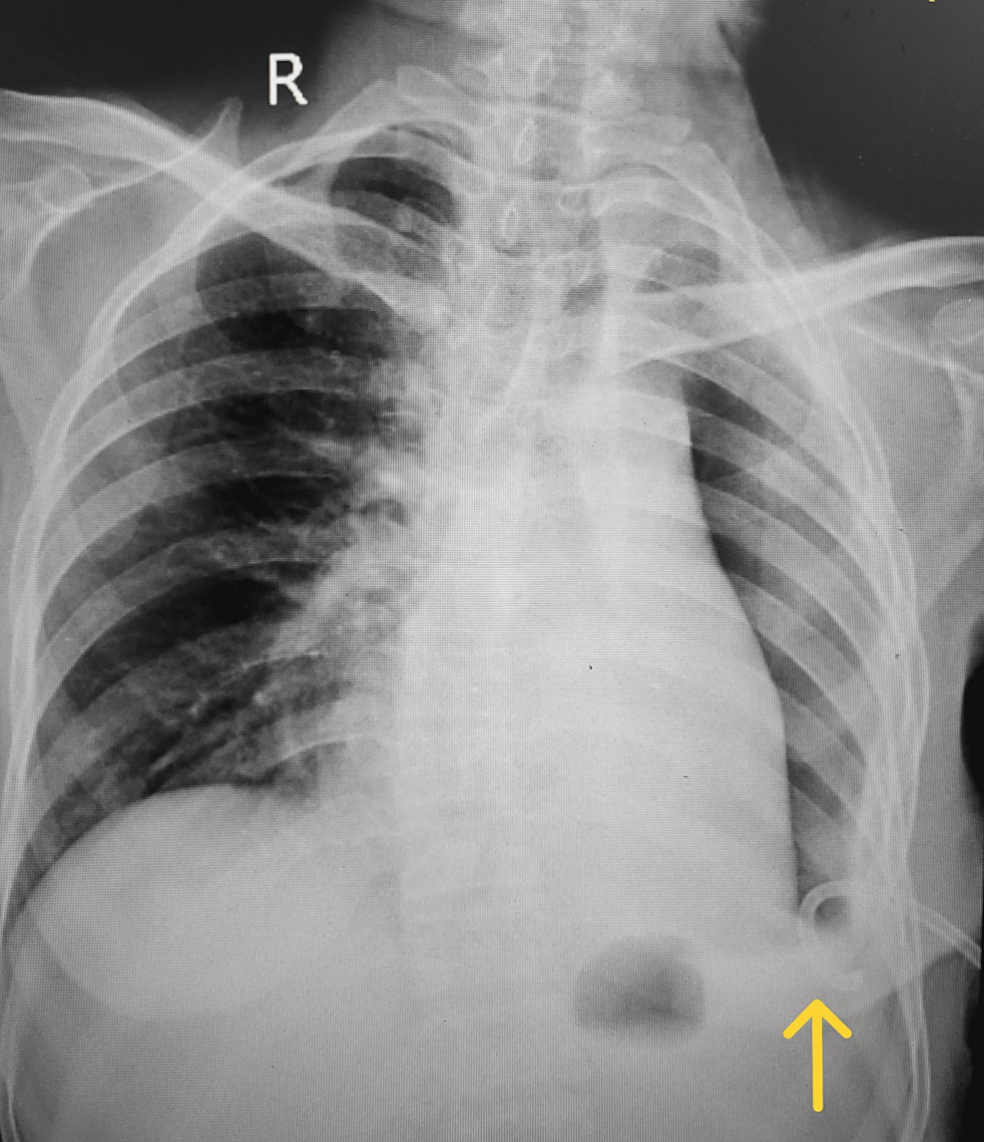
A complete destruction and resolution of the cysts on the left hemithorax. The underlying lung remained collapsed with a non-resolving pyopneumothorax with a pigtail chest drain (yellow arrow) in situ on the left side.

During the course of the hospital stay, approximately 4-5 liters of fluid was drained. A bronchoscopy was also done, which revealed no endobronchial cysts or abnormality. Ultrasound of the abdomen excluded the presence of any other similar lesion in the abdominopelvic region. The patient was then advised to take oral albendazole 400 mg twice daily, and anti-tubercular treatment was stopped. Later, all the cysts disappeared remarkably just by continuing medical treatment (Figure 5).

The patient had also developed secondary bacterial pyopneumothorax with a persistent air leak from the bronchopleural fistula and hence was referred to a thoracic surgeon for surgical management. The patient is now symptomatically better and continued on conservative management of oral albendazole for six months, with a pigtail chest tube kept in situ, and has been planned for surgery.

## Discussion

Farmers, animal keepers, and shepherds are at a higher risk of echinococcosis because of close proximity to animals and might remain asymptomatic. The rupture of hydatid cysts remains a serious and fatal outcome, which can even happen during any intervention.

According to an interesting theory, hydatid can spread through the ascending diaphragmatic lymphatic drainage system along the internal mammary nodes anteriorly and the intercostal nodes posteriorly in cases of intrathoracic extrapulmonary hydatid illness. Additionally, hematological dissemination may happen through an arterial branch of the thoracic aorta, such as the diaphragmatic or intercostal arteries [3,4].

*Echinococcus* can affect the thorax at numerous sites, the lungs being the most common, followed by the pleura, mediastinum, chest wall, pericardium, and diaphragm. There could be a diagnostic challenge, and the available treatment options may be affected by the unusual complications or unusual locations of the disease; when dealing with patients who have lived in endemic areas or who exhibit symptoms of hydatid illness elsewhere in the body, radiologists and clinicians should consider hydatid disease [5].

The most prevalent complication of pulmonary hydatid disease, and also a complex cyst, is the cyst rupture into a bronchus. Life-threatening complications may arise if a cyst bursts into the pleural or cardiac cavity. This might result in life-threatening anaphylaxis, spontaneous pneumothorax or hydropneumothorax, and sometimes even recurrent pneumothorax [6-8].

In regions where hydatid cysts are common, a potential diagnosis is considered when individuals have lung cysts and a background of contact with sheep and dogs. Radiological and serological tests are the primary methods employed for validating the diagnosis. Fewer than a quarter of those affected exhibit an increase in eosinophils in their peripheral blood, along with leukocytosis and a raised erythrocyte sedimentation rate which are more common in cases with ruptured hydatid cysts. Nonetheless, these standard examinations lack specificity and can show elevated results in numerous different situations [9,10].

## Conclusions

A massive pleural effusion, dyspnea, and fever can be symptoms of intrathoracic extrapulmonary hydatidosis which should be suspected radiologically and confirmed microbiologically or operatively. Notably, in our particular case, the pleural fluid displayed an elevated ADA level, which prompted the initiation of anti-tubercular treatment for the patient. However, it's not widely recognized that parasitic pleural effusions can lead to an increase in ADA levels. Further research and evidence are required for us to make definitive statements in this regard.

While medical treatment has demonstrated its effectiveness in our case, surgical intervention remains the preferred approach for the complete removal of intrathoracic cysts. This case report underscores the potential presence of a zoonotic lung and pleura ailment as a contributing factor to pleural effusion or pyopneumothorax, thus warranting its inclusion among the possible causes to be considered.
